# Characterization and genomic analysis of an oceanic cyanophage infecting marine *Synechococcus* reveal a novel genus

**DOI:** 10.3389/fmicb.2023.1231279

**Published:** 2023-08-04

**Authors:** Tiancong Wang, Lin Luo, Yao Xiong, Chuxiao Wang, Hongbing Shao, Min Wang, Cui Guo

**Affiliations:** ^1^College of Marine Life Sciences, Frontiers Science Center for Deep Ocean Multispheres and Earth System, Institute of Evolution and Marine Biodiversity, Ocean University of China, Qingdao, China; ^2^UMT-OUC Joint Centre for Marine Studies, Ocean University of China, Qingdao, China; ^3^Haide College, Ocean University of China, Qingdao, China; ^4^The Affiliated Hospital of Qingdao University, Qingdao, China

**Keywords:** cyanophage, *Synechococcus*, *Kyanoviridae*, novel genus, auxiliary metabolic genes, genomic and phylogenetic analysis, biogeographical distribution

## Abstract

Cyanophages play a crucial role in the biogeochemical cycles of aquatic ecosystems by affecting the population dynamics and community structure of cyanobacteria. In this study, a novel cyanophage, *Nanhaivirus ms29*, that infects *Synechococcus* sp. MW02 was isolated from the ocean basin in the South China Sea. It was identified as a T4-like phage using transmission electron microscopy. Phylogenetic analysis demonstrated that this cyanophage is distinct from other known T4-like cyanophage, belonging to a novel genus named *Nanhaivirus* within the family *Kyanoviridae*, according to the most recent classification proposed by the International Committee on Taxonomy of Viruses (ICTV). The genome of this novel cyanophage is composed of 178,866 bp of double-stranded DNA with a G + C content of 42.5%. It contains 217 potential open reading frames (ORFs) and 6 tRNAs. As many as 30 auxiliary metabolic genes (AMGs) were identified in the genome, which related to photosynthesis, carbon metabolism, nutrient uptake and stress tolerance, possibly reflecting a genomic adaption to the oligotrophic environment. Read-mapping analysis showed that *Nanhaivirus ms29* mainly distributed in temperate and tropical epipelagic waters. This study enriches of the virus gene database of cyanophages and provides valuable insights into the phylogeny of cyanophages and their interactions with their hosts.

## 1. Introduction

Marine unicellular cyanobacteria, including *Prochlorococcus* and *Synechococcus*, are among the oldest photosynthetic autotrophic organisms on Earth, and plays an important role in shaping the global biogeochemistry with their high abundance and key metabolic activities (Scanlan et al., 2009). *Synechococcus*, in particular, is widely distributed with the annual mean global abundances of ~7.0 × 10^26^ cells, contributing to around 16.7% of the ocean’s net primary production (Waterbury et al., 1986; Flombaum et al., 2013). It exhibit a remarkable genetic diversity and comprise at least 20 distinct phylogenetic lineages, as evidenced by molecular markers (Scanlan, 2003; Mazard et al., 2012). Based on the phylogenetic analysis, marine *Synechococcus* strains form a distinct branch known as cluster 5, which is divided into three subclusters: 5.1, 5.2, and 5.3. Among them, subgroup 5.1 is the most abundant and diverse subgroup in the marine environment, further classified into at least nine clades (Fuller et al., 2003; Xia et al., 2015). Certain strains of *Synechococcus* are known to exhibit niche preferences, such as temperature, light and salinity, and therefore show different geographical distributions (Pittera et al., 2014). However, the population dynamics of cyanobacteria can be influenced by infection of cyanophages, leading to changes in the structure and function of the marine microbial food web.

Cyanophages constitute a genetically diverse and highly abundant component of marine planktonic communities. They play a crucial role in regulating the abundance, diversity and function of cyanobacterial communities. It has been reported that these phages can cause mortality rates ranging from 0.005% to 30% of their cyanobacterial hosts on a daily basis (Waterbury and Valois, 1993). Moreover, during phage infection of their hosts, gene transfer and recombination events, i.e., horizontal transfer of auxiliary metabolic genes (AMGs), often occur, which can continuously influence their evolution in a long-term phage-host symbiotic environment (Sullivan et al., 2010; Chevallereau et al., 2022). These AMGs encode proteins involved in host photosynthesis, carbon metabolism, nucleotide biosynthesis, stress responses, and nutrient utilization (Ignacio-Espinoza and Sullivan, 2012; Fuchsman et al., 2021), and thus allow the cyanophage to drive coevolution with their hosts and achieve effective replication of progeny by providing complementary support during infection (Hurwitz et al., 2015).

Since publication of the first complete genome sequence of cyanophage P60 infected marine *Synechococcus* in 2002 (Chen and Jingrang, 2002), there has been an increasing number of cyanophage isolates, which largely enriched the genomic database and provided valuable insights into their biology and ecology. Nevertheless, compared with the number of phage sequences obtained by using metagenomic techniques, the available cyanophage isolates still represent a limited fraction, estimated to be only 1% to 5% of the total number of cyanophages in the ocean (Simmonds et al., 2017). Furthermore, most of the confirmed T4-like cyanophage isolates are from coastal estuary regions, while only about 20% originating from the open ocean areas (Jiang et al., 2020; Zhang et al., 2020). This highlights the possibility that our comprehension of cyanophages could be limited to particular geographical areas, indicating a potential for greater diversity in cyanophage genomes and behaviors yet to be uncovered across various marine habitats. In this study, a novel marine cyanophage, *Nanhaivirus ms29*, which infects *Synechococcus* MW02, was isolated from an oligotrophic open-ocean site in the South China Sea. Its morphological, physiological and genomic features were characterized. Comparative genomics, phylogenetic analysis, and metagenomic read-mapping were applied to investigate the evolution, taxonomy, and biogeography of this cyanophage, thereby expanding the database of the cyanophage distributed in the oligotrophic environment. This study contributes to our knowledge of phage-host interactions and their adaptation to the marine environment, shedding light on the ecological and evolutionary dynamics of cyanophages in the open ocean.

## 2. Materials and methods

### 2.1. Cyanophage isolation

**Figure 1:**
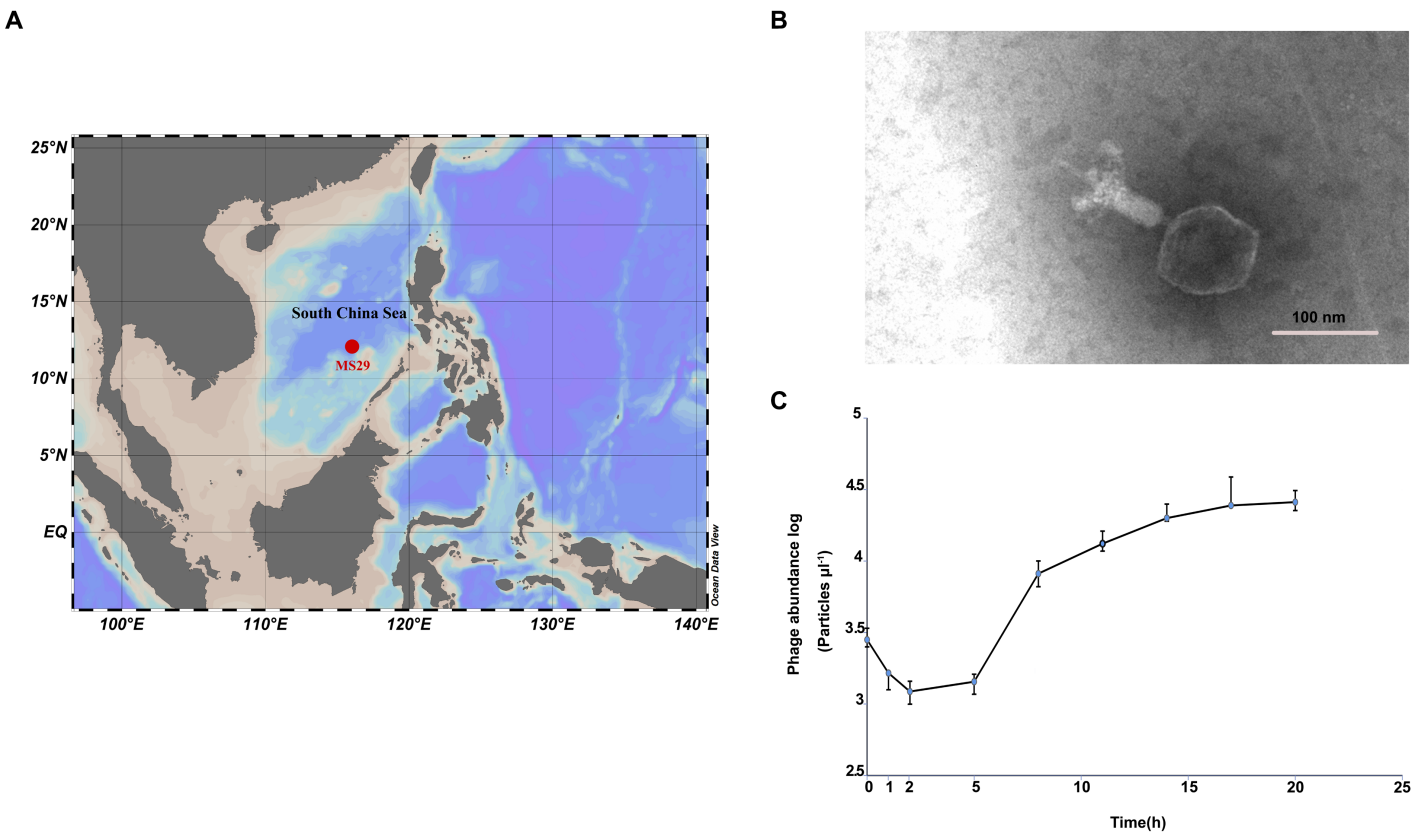
**(A)** The isolation site of *Nanhaivirus ms29* in the South China Sea. **(B)** Transmission electron micrograph of *Nanhaivirus ms29*. **(C)** One-step growth curve of *Nanhaivirus ms29*.

Surface seawater (150 L) was collected from an oligotrophic ocean basin site MS-29 (12°0.0′ N, 116°0.0′ E) with a bottom depth of 4,079 m (Figure 1A). The collected seawater was filtered sequentially through 3-μm and 0.22-μm polycarbonate membranes (Isopore™, Millipore). The filtrate was concentrated by tangential flow filtration using a 50 kDa cartridge (Pellicon® XL Cassette, Biomax® 50 kDa; polyethersulfone, Millipore, United States) to increase the viral concentration by 300 times the initial concentration. The viral concentrate was stored at 4°C in the dark.

The host of the cyanophages is *Synechococcus* sp. strain MW02 (NCBI accession number KP113680) belonging to clade IX, which belongs to clade IX in subcluster 5.1 and was originally isolated from the Hong Kong coastal waters impacted by various water masses (Xia et al., 2015). This clade appears to have a high tolerance to different environmental conditions and is capable of adapting to diverse ecological niches (Xia et al., 2015, 2021). The algal culture was grown in conical flasks with f/2 seawater medium under a constant illumination of approximately 25 μmol m^−2^ s^−1^ at 25°C in a 12-h/12-h light–dark cycle. Liquid infection was used to isolate the cyanophage, where aliquots of virus concentrate were added to the exponentially growing host culture in a ratio of 1:9. Phage-host suspensions were incubated for about 1 week until lysed host cells were observed based on the color and turbidity of the lysate. A separate control group was set up with pure algal culture.

### 2.2. Phage purification

The cyanophage obtained in this study was purified using a serial dilution method followed by sucrose density gradient centrifugation (Jiang et al., 2020). To begin with, the lysate was diluted at a series levels (10-fold dilution over 7 orders of magnitude) using sodium chloride-magnesium sulfate (SM) buffer (100 mM NaCl, 50 mM Tris, 10 mM MgSO_4_, and 0.01% gelatin, pH 7.5). The infectivity of each dilution was tested, and the most diluted sample that induced lysis was selected for next round of continuous dilution and infection assay using the same method. After three rounds of purification, a pure lysate containing a single phage strain was obtained theoretically (Wilhelm et al., 2006). The obtained cyanophage was concentrated using high recovery centrifugal filter units (Amicon® Ultra-15, MWCO 30 kDa, Millipore; Chénard and Suttle, 2008).

The concentrated phage suspensions were further purified by sucrose density gradient centrifugation. Four sucrose gradients of 20%, 30%, 40%, and 50% (w/v) were prepared, and the suspensions were centrifuged at 110,000 ×g for 2.5 h at 4°C in a Beckman-Coulter ultra-high-speed centrifuge. A visible band between the 40 and 50% gradient was carefully extracted using a syringe with a needle and diluted in TE buffer. The dilution was then centrifuged at 110,000 ×g at 4°C for 3 h to obtain the concentrated cyanophages. The purified phage precipitates were resuspended in SM buffer and stored at 4°C.

### 2.3. Morphological examination by transmission electron microscopy

The purified phage suspension (20 μL) was placed onto a 200-mesh copper grid. A drop of 1% (w/v) phosphotungstic acid (pH 7.2) was added onto the grid, covering the phage particles, and allowed to stain for 10 min (as described by Wilson et al., 1993). After staining, the excess stain was blotted off gently with filter paper. The stained phage particles on the grid were then observed using a transmission electron microscope (JEOL JEM-1200EX, Japan) operating at 100 kV.

### 2.4. One-step growth curve and host specificity test

To understand the growth kinetics of *Nanhaivirus ms29* in its host, a one-step growth curve was conducted. Briefly, exponentially growing *Synechococcus* sp. strain MW02 were inoculated in medium. Cyanophage suspension was added to the host culture at a multiplicity of infection (MOI) of 0.1 to increase the chance of infection for the host cells during the first infection cycle. The samples were collected at various time points, including 0, 1, 2, 5, 8, 11, 14, 17, and 20 h after infection. The abundance of phage and host was measured using flow cytometry (McSharry, 2000).

The infectivity of *Nanhaivirus ms29* was tested using nine *Synechococcus* strains, including *Synechococcus* WH7803, WH8102, MW02, MW03, LTWRed, LTWGreen, PSHK05, CCMP1333, and PCC7002. The cyanophage solution was added to each host *Synechococcus* culture in logarithmic growth phase at a volume ratio of 1:9, in triplicates. The cyanophage solution was replaced by the medium in the control group. The mixtures were incubated under the same conditions described above. Cell lysis was monitored and compared in the control and cyanophage solution groups every day for 2 weeks to examine the infectivity.

### 2.5. Phage DNA extraction, sequencing, and annotation

Phage genomic DNA was extracted using the TIANamp Virus DNA Kit (TIANGEN) and sequenced using Illumina Miseq 2 × 300 paired-end sequence method. Raw reads were cleaned up by trimming adaptor sequences and removing low-quality reads. Reads containing >40% low-quality bases (mass value ≤ 20), >10% N content, and overlap with the adapter for >15 bp with less than 3 mismatches, were removed. Clean reads were then assembled into a single contig using Velvet (Version 1.2.07). The GapCloser (v1.12) was applied to fill the gap in preliminary assembly results and the fragments shorter than 500 bp were eliminated. The complete genome of *Nanhaivirus ms29* has been deposited in the GenBank database under the accession number OP807803. The prediction of the hypothetical open reading frames (ORFs) was performed with Prodigal (version 2.6.3; Hyatt et al., 2010). The prediction of the transfer RNA (tRNA) genes sequences was performed using the RAST online server^1^ (Brettin et al., 2015). After that, the translated amino acid sequences were scanned for homologs by BLASTp (version 2.9.0) in Non-Redundant (nr) protein database^2^ of the GenBank. The protein domains were predicted and analyzed by Pfam database^3^ (Hofle and Brettar, 1996), and Kyoto Encyclopedia of Genes and Genomes (KEGG) database.^4^ The genome information of all *Kyanoviridae* members was downloaded from the NCBI database, and the same steps as above were used to annotate the genome, the content of AMGs in *Nanhaivirus ms29* and member genomes was determined.

### 2.6. Phylogenetic analysis

To classify *Nanhaivirus ms29*, 57 isolated reference genome sequences of the *Kyanoviridae* were downloaded from the NCBI Virus database, and 56 complete genome sequences were selected for analysis. A proteome clustering tree based on genome-wide sequence similarities computed by tBLASTx was generated with the genomes of *Nanhaivirus ms29* and the other 56 selected cyanophages using ViPTree online server (Nishimura et al., 2017). The conserved portal protein sequences were used to construct a single gene phylogenetic tree (Zhong et al., 2002; Weinheimer and Aylward, 2022). These specific amino acid sequences were aligned with MEGA (version 7.0.18) and trimmed with TrimAl (version 1.4.15). The maximum-likelihood (ML) phylogenetic tree was constructed using IQ-tree (multicore version 2.0.3), and the bootstrap values were based on 1,000 replicates (Nguyen et al., 2015). The phylogenetic tree was visualized with iTOL.^5^

### 2.7. Global distribution and relative abundance of *Nanhaivirus ms29*

To determine the relative abundances of *Nanhaivirus ms29*, RPKM (Reads Per Kilobase per Million mapped reads) was calculated using CoverM (v0.6.0).^6^ The *Nanhaivirus ms29* contigs were mapped to quality-controlled reads from the 154 Global Ocean Viromes (GOV 2.0) dataset using the minimap2 (v2.17) program (Li et al., 2010, 2021). To compare the abundance distribution of this novel cyanophage with other T4-like cyanophages in global oceans, 45 cyanophage sequences from different cyanophage genera in *Kyanoviridae* were selected as references. The relative abundance of *Nanhaivirus ms29* was compared among five marine viral ecological zones (VEZs) defined by the Global Ocean Viromes (GOV 2.0) dataset: Arctic (ARC), Antarctic (ANT), temperate and tropical epipelagic (EPI), temperate and tropical mesopelagic (MES), and bathypelagic (BATHY; Gregory et al., 2019). To map the global distribution of *Synechococcus* phage *Nanhaivirus ms29*, the relative abundance was log_10_(x + 1) transformed and visualized using a heatmap.

## 3. Results and discussion

### 3.1. Morphology and biological characterization of *Nanhaivirus ms29*

Transmission electron microscopy examination showed that *Nanhaivirus ms29* exhibited a T4-like cyanophage with an icosahedral head (approximately 89.945 nm in diameter) and a long straight contractile tail (length 124.166 nm; Figure 1B). The one-step growth curve showed that *Nanhaivirus ms29* had an adsorption period of 1–2 h, followed by a latent period of 2–5 h (Figure 1C). The first peak release time occurred approximately 17 h after inoculation. The infection cycle of this cyanophage is similar to that of the cyanophage *Ormenosvirus syn9* (Doron et al., 2016), which is its closest relative on the protein taxonomic tree. Host specificity test showed that *Nanhaivirus ms29* could not infect the other 9 strains of *Synechococcus.*

### 3.2. Phylogenetic analyses and taxonomy—proposal of a new cyanophage genus

**Figure 2:**
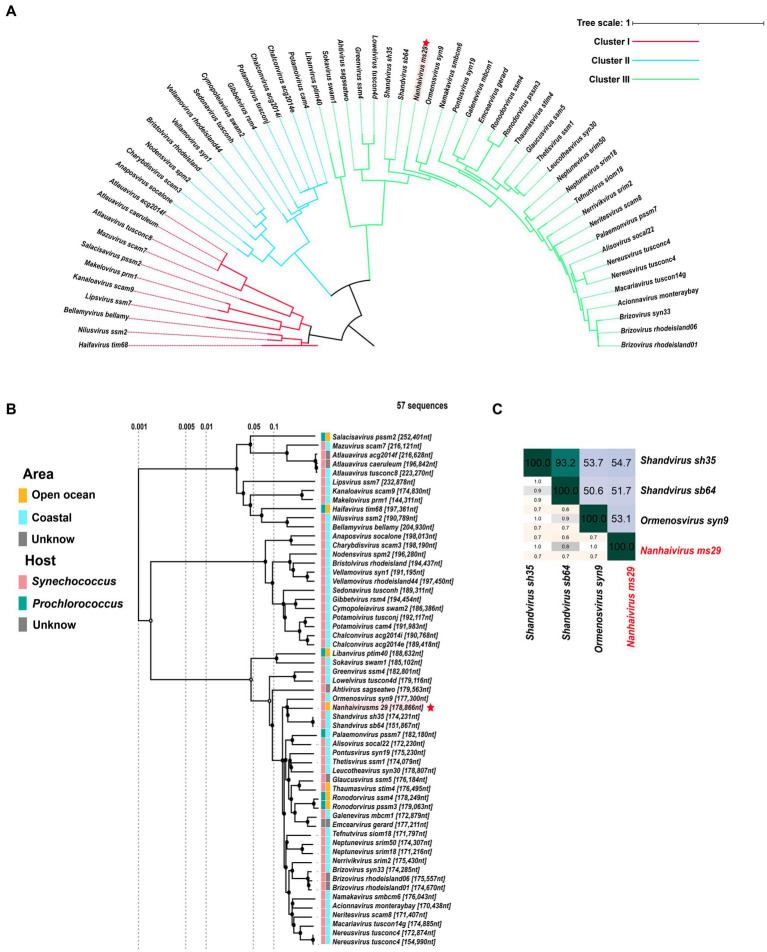
**(A)** The maximum-likelihood phylogenomic tree based on g20 gene that encodes the portal vertex protein. **(B)** The proteome cluster tree of *Nanhaivirus ms29* with members in *Kyanoviridae*. **(C)** Heatmap of average nucleotide identity (ANI) identity between *Nanhaivirus ms29* and its close relatives.

In the new virus classification system proposed by ICTV in October 2020, T4-like cyanophages were grouped under the family *Kyanoviridae*. So far, this family has been classified into 45 genera and 56 species. However, it appears that this proposal has not gained widespread adoption and many studies still uses the previous classification method. Here, we used genome sequences of *Nanhaivirus ms29* and other 56 representative *Kyanoviridae* isolates to perform phylogenetic analysis. Newly identified cyanophages were usually classified using phylogenetic trees constructed from viral marker genes such as portal proteins, major capsid proteins, and large subunits of terminases (Dion et al., 2020). Here, we used the portal-protein-encoding homolog, gene 20 (g20), to construct a single-gene phylogenetic tree of 57 phages (Figure 2A). This approach has been previously used to classify T4-like phages (Zhong et al., 2002). Based on the results of the phylogenetic tree, the 57 cyanophages strains were classified into three clusters: Cluster I, Cluster II and Cluster III. The *Nanhaivirus ms29* is clustered separately in Cluster III, forming a distinct branch. To further explore the phylogenetic relationship between *Nanhaivirus ms29* and the other T4-like cyanophages and evaluate their taxonomic position, we constructed a genome-wide proteomic tree (Figure 2B). The result showed that *Ormenosvirus syn9*, *Shandvirus sh35* and *Shandvirus sb64* were most closely related to *Nanhaivirus ms29* in the proteome tree. *Shandvirus sh35* and *Shandvirus sb64* infect *Synechococcus* sp. WH8102 and were isolated from the Yellow and Bohai estuaries in China (You et al., 2019), while *Ormenosvirus syn9* was isolated from Woods Hole Harbor in the United States and also hosted by *Synechococcus* sp. WH8102 (Weigele et al., 2007). *Nanhaivirus ms29* is the first cyanophage isolated from the open ocean to be included in this little clade and to infect *Synechococcus* MW02. According to the nucleotide homology heat map of 57 cyanophages, *Shandvirus sh35* had the highest nucleotide identity with *Nanhaivirus ms29*, but the observed similarity was only 54.7% (Figure 2C). Therefore, according to the latest genus classification criteria proposed by ICTV (nucleotide homology with existing members < 70%), we proposed that the newly discovered *Synechococcus* phage represents a new cyanophage genus in *Kyanoviridae* and named it *Nanhaivirus*. The new genus *Nanhaivirus* has been submitted to ICTV.

### 3.3. Genomic features

**Figure 3:**
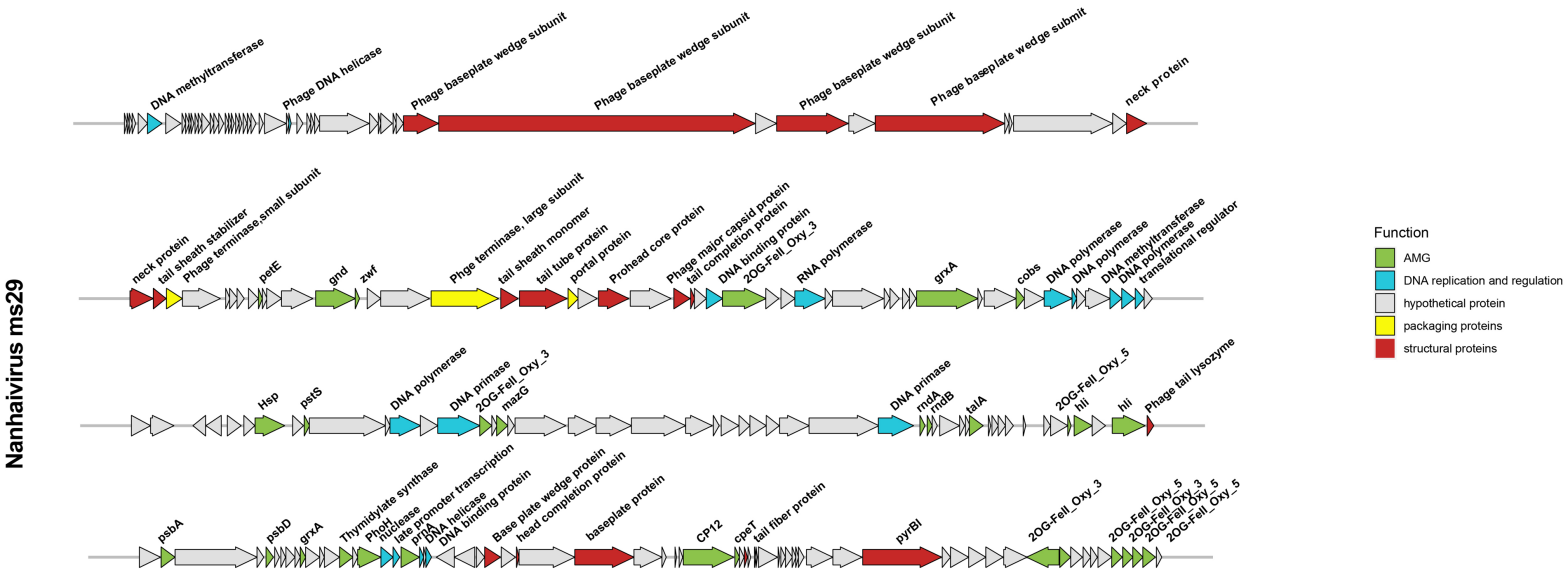
Genome map of *Nanhaivirus ms29* and functional annotation of the predicted proteins.

The genome of *Nanhaivirus ms29* is a linear double-stranded DNA of 178,866 bp with a GC content of 42.5%. The GC content of most *Synechococcus* phages genomes typically ranges from 35.4% to 51.7% (Jiang et al., 2020). It has been shown that organisms such as bacteria, phages, and plasmids, which are dependent on parasitism for survival, tend to have low G + C content in their genomes (Rocha and Danchin, 2002), probably due to differences in the cost of relevant metabolites in cells and the limited availability of G and C relative to A and T/U (Rocha and Danchin, 2002). The *Nanhaivirus ms29* contains 217 ORFs and six different tRNA types that encode for 5 amino acids (Ser, Arg, Val, Pro, Gly). After comparison in the NCBI non-redundant (NR) protein database, Pfam protein database, and KEGG protein database, 67 out of 217 ORFs (30.87%) matched with homologs of other known functional proteins, while the remaining 150 ORFs (69.13%) were predicted to be hypothetical proteins due to the lack of relevant genetic information (Figure 3). The predicted functions of these 67 ORFs included structuring (18 ORFs), DNA replication and regulation (16 ORFs), auxiliary metabolic genes (AMGs, 30 ORFs), and packaging proteins (3 ORFs).

The structural proteins of *Nanhaivirus ms29* include the neck protein (ORF 50, 51), prohead core protein (ORF 72), baseplate protein (ORF 40, 41, 43, 45, 176, 180, 188), tail tube protein (ORF 69, 150), tail fiber protein (ORF 201), phage tail sheath protein (ORF 52, 68), head completion protein (ORF 178), phage tail completion protein (ORF 75), and the major capsid proteins (ORF 74). The large and small terminase subunits (ORF 67 and ORF 53) plus the portal protein (ORF 70) belong to the packaging module of *Nanhaivirus ms29* (Fujisawa and Morita, 1997). The DNA replication and regulation module consist of 16 ORFs, including four DNA polymerase-associated proteins (ORF 93, 94, 98, 112), DNA methyltransferase (ORF 6, 112), DNA primase (ORF 114, 131), DNA helicase (ORF 29, 170), DNA binding protein (ORF 77, 172), transcriptional regulator (ORF 99, 168) and RNA polymerase (ORF 81), nuclease (ORF 167). These genes, which are directly involved in DNA replication and transcription, are ubiquitous in most living organisms (Stubbe, 2000).

### 3.4. Large numbers of AMGs to enhance the fitness of *Nanhaivirus ms29* in the oligotrophic environment

**Figure 4:**
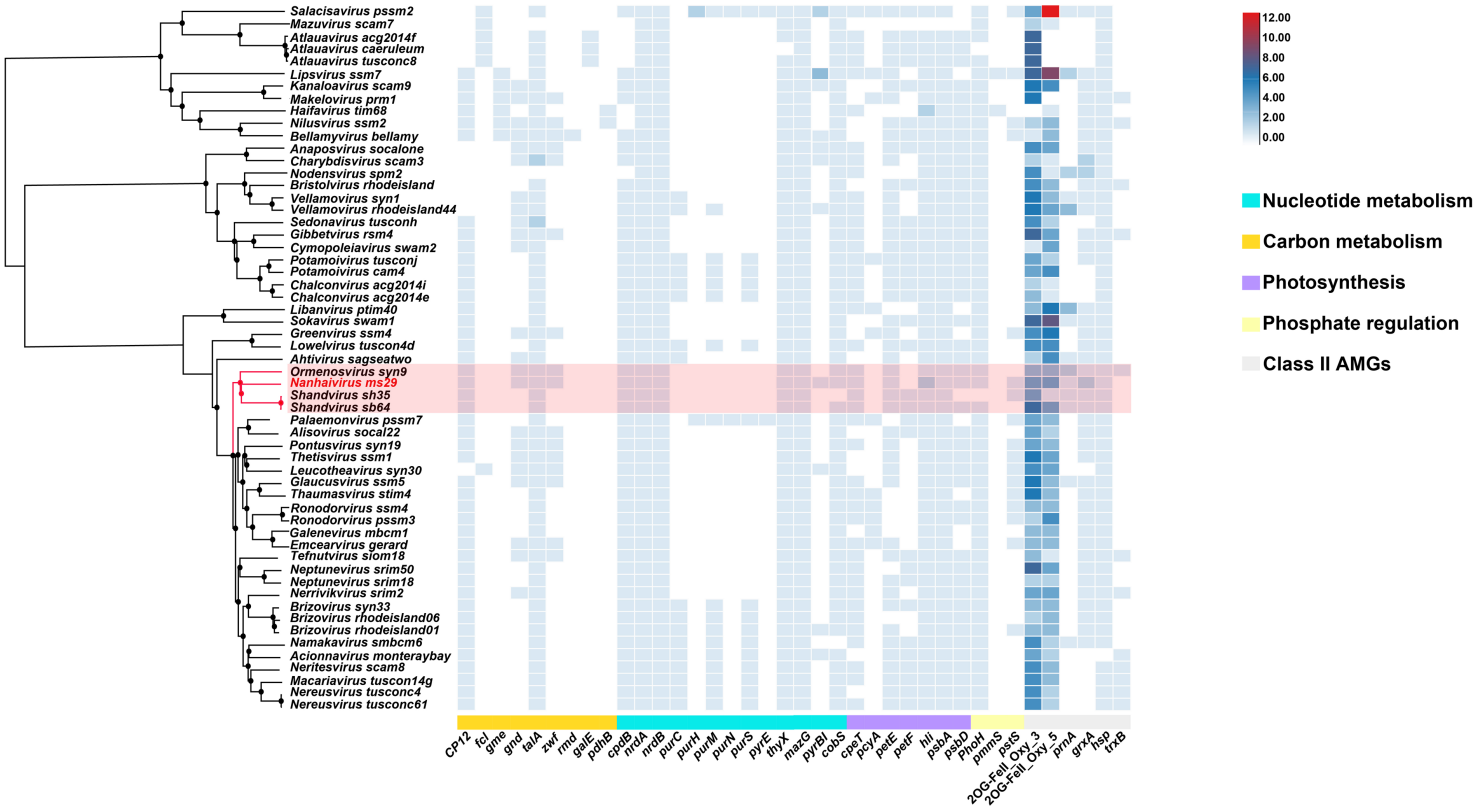
AMGs copy number heat map in 57 viral genomes. The genome-wide phylogenetic tree of the 57 viruses is shown next to the viral name. *CP12*, Calvin cycle Inhibitor; *fcl*, GDP-L-fucose synthase; *gme*, GDP-D-mannose 3′, 5′-epimerase; *gnd*, 6-phosphogluconate dehydrogenase; *talA*, Transaldolase; *zwf*, Glucose-6-phosphate 1-dehydrogenase; *rmd*, GDP-4-dehydro-6-deoxy-D-mannose reductase; *galE*, UDP-glucose 4-epimerase; *pdhB*, Pyruvate dehydrogenase; *cpdB*, 2′,3′-cyclic-nucleotide 2′-phosphodiesterase; *nrdA*, Ribonucleoside-diphosphate reductase alpha chain; *nrdB*, Ribonucleoside-diphosphate reductase beta chain; *purC*, Phosphoribosylaminoimidazole-succinocarboxamide synthase; *purH*, Phosphoribosylaminoimidazolecarboxamide formyltransferase; *purM*, Phosphoribosylformylglycinamidine cyclo-ligase; *purN*, Phosphoribosylaminoimidazolecarboxamide formyltransferase; *purS*, Phosphoribosylformylglycinamidine synthase subunit; *purE*, 5-(carboxyamino) imidazole ribonucleotide mutase; *thyX*, Thymidylate synthase; *MazG*, Nucleoside triphosphate diphosphatase; *pyrBI*, Carbamoyltransferase; *cpeT*, Photosynthesis-antenna proteins; *pcyA*, Phycocyanobilin:ferredoxin oxidoreductase; *petF*, Ferredoxin; *petE*, Plastocyanin; hli, Highlight inducing protein; *psbA*, Photosystem II reaction center D1 protein; *psbD*, Photosystem II reaction center D2 protein; *phoH*, Phosphohistidine phosphatase; *pmmS*, 2-phosphinomethylmalate synthase; *pstS*, Phosphate transport system substrate-binding protein; *prnA*, Tryptophan 7-halogenase; *cobS*, Cobaltochelatase; *grxA*, Glutaredoxin; *hsp.,* Heat shock protein; *trxB*, Thioredoxin; 2OG-FeII_Oxy_3 and 2OG-FeII_Oxy_5 are different subfamilies within the 2OG-Fe(II) oxygenase superfamily.

Autonomous replication of viruses inside host cells leads to the expression of viral genes, including AMGs, which are associated with a variety of physiological activities in virus-infected hosts, including photosynthesis (Mann et al., 2003), carbon metabolism (Thompson et al., 2011), and nucleotide metabolism (Dwivedi et al., 2013). Recently, Class II AMGs, which do not exist in the KEGG database or lack explicit annotations of metabolic pathways, were also suggested to be important in cyanophage function (Hurwitz and U’Ren, 2016). In the genome of *Nanhaivirus ms29*, 30 AMGs were identified, including 26 Class I AMGs and 4 Class II AMGs (Figure 4). AMGs involved in photosynthesis (i.e., *petE*, *hli*, *psbA*, *psbD*, *cpeT*) may play an important role in maintaining the photosynthetic activity of infected host cells, providing energy for phage replication and increasing phage fitness (Lindell et al., 2005). Genes related to carbon metabolism included *CP12*, *gnd*, *zwf*, and *talA*. Previous studies have demonstrated that phage-encoded *CP12* and *talA* can redirect the energy flow of ATP and NADPH produced during photosynthesis away from the Calvin cycle toward the biosynthesis of dNTPs for phage replication during host infection (Thompson et al., 2011). AMGs related to phosphate regulation (*pstS*, *phoH*) may involve in phosphate uptake and starvation. Nucleotide metabolism (*MazG*, *nrdA*, *nrdB*, *thyX*, *pyrBI*, *cobS*) are genes related to genome replication and transcription in cyanophages. For example, the phage-encoded *MazG* was proposed to regulate the cellular level of ppGpp and therefore affect transcription and translation in the host and extend the period of cell survival under the stress of phage infection (Clokie and Mann, 2006). In addition, Class II AMGs (ORF 78, 115, 146, 208, 213, 214, 215, 216, 2OG-Fe(II)_Oxy, ORF 88, 160 *grxA*, ORF 107 *hsp.,* ORF 169 *prnA*) were also identified in genome of *Nanhaivirus ms29.* For example, the 2OG-Fe(II) oxygenase superfamily proteins have the highest copy number among all AMGs in the genome of *Nanhaivirus ms29*, which may involve in performing multiple functions (van Staalduinen and Jia, 2014).

To compare the distribution of different AMGs present in the genomes of representative T4-like cyanophages, a quantitative heatmap was constructed and the cyanophages were clustered based on their proteome (Figure 4). Among the 57 phages compared, the genome of *Nanhaivirus ms29* contains a higher number of AMGs, exceeds approximately 85% of the phages analyzed. It has been suggested that the AMG repertoire of virus is correlated with its isolated environment (coastal vs. open ocean; Crummett et al., 2016). The presence of relatively large number of AMGs in the genome of *Nanhaivirus ms29*, which was isolated from open ocean, may indicate its coping strategies and adaptive evolution toward oligotrophic environments.

In addition, we observed that that the clade composed of *Nanhaivirus ms29* and three other cyanophages exhibit a similar AMG composition, with only minor differences for some low-frequency AMGs among the clades, such as *gnd*, *zwf*, *pyrBI*, and *pstS*. To acquire phosphorus, cyanobacteria typically use an ABC-type phosphate transporter, consisting of a high affinity phosphate-binding protein (*pstS*) and two transmembrane proteins (*pstC* and *pstA*; Kamennaya et al., 2020). Studies have shown that the *pstS* gene in cyanophages can be effectively expressed during host infection and improve the phosphate uptake rate of the host after infection (Zhao et al., 2022), which may contribute to adaptation of the cyanophage to the oligotrophic environment. Coincide with our observation, *pstS* has been suggested to be one of the genes contributing to the difference in AMG content between coastal and open ocean phage isolates (Fuchsman et al., 2023). The enzymes produced by the *gnd* and *zwf* genes in cyanophages are involved in the pentose phosphate pathway, an important metabolic pathway providing reducing capacity and the carbon skeleton required for biosynthesis (Thompson et al., 2011). The enzymes produced by these two genes can convert 6-phosphogluconate to NADPH and ketones. This process could be important for the growth and replication of oligotrophic cyanophages, as NADPH is a crucial cofactor needed for energy production and biosynthesis (Doron et al., 2016). *PyrBI* that encodes catalytic subunit of aspartate carbamoyltransferase catalyzes the first step in the pyrimidine biosynthetic pathway. However, the role of *pyrBI* in cyanophages is still not clear. In brief, variation in content of AMGs among closely related cyanophages reflect the different horizontal evolutionary paths resulting from selection under diverse environmental pressures during vertical evolution of homologous cyanophages. Therefore, further research on AMGs among cyanophages of different habitats will deepen our understanding of cyanophage-host interactions in the environment.

### 3.5. Ecological distribution of *Nanhaivirus ms29*

**Figure 5:**
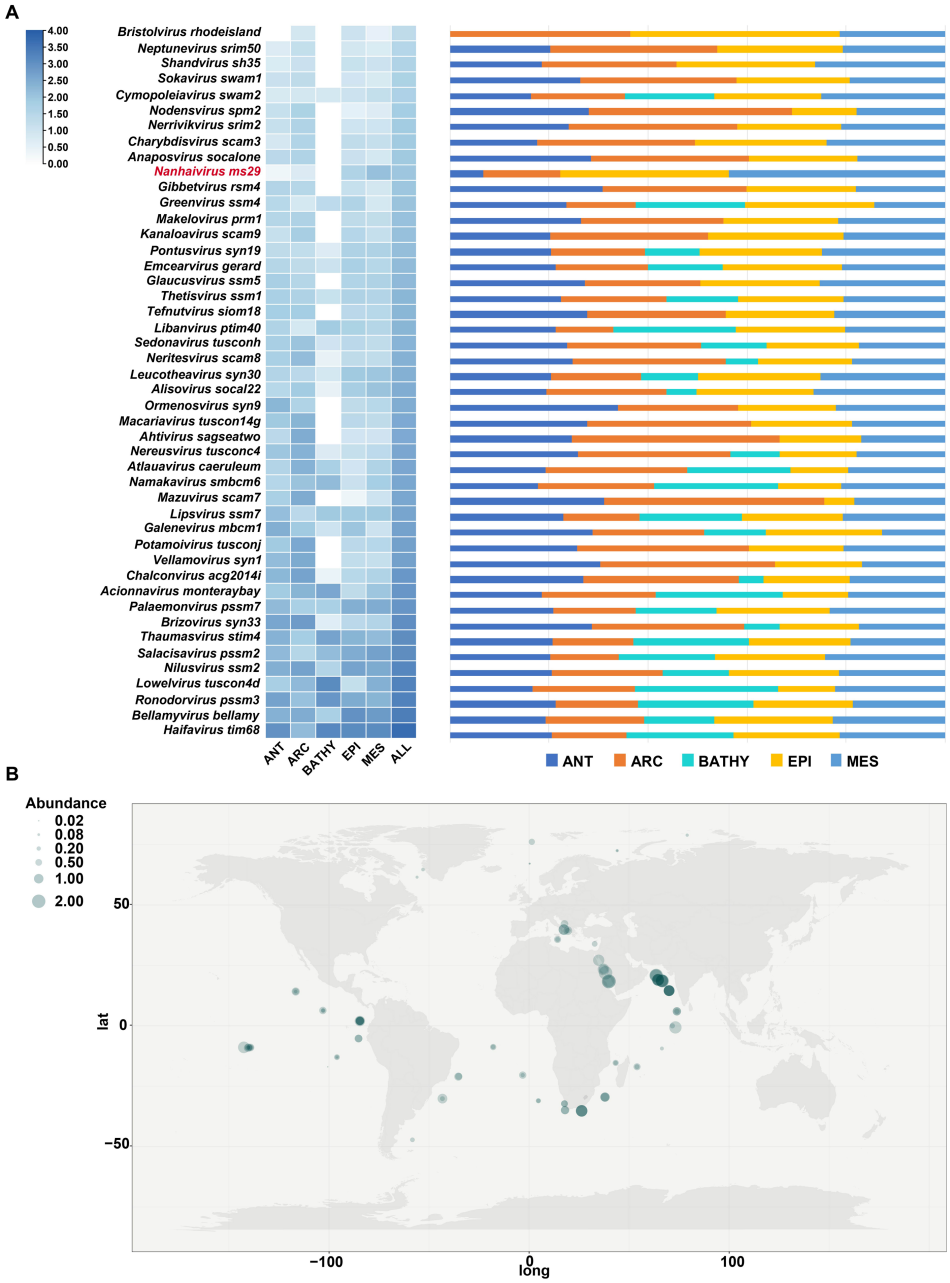
Relative abundance of *Nanhaivirus ms29* in marine environments. **(A)** The relative abundance of *Nanhaivirus ms29* and other members in *Kyanoviridae* family calculated in 154 viromes from the Global Ocean Viromes (GOV 2.0) data set. The abundance was expressed as RPKM (Reads Per Kilobase per Million mapped reads) and calculated using CoverM. The values were normalized by the number of databases in each viral ecological zone (VEZ) and transformed by log10 (X + 1). The five marine VEZs are Arctic (ARC), Antarctic (ANT), temperate and tropical epipelagic (EPI), temperate and tropical mesopelagic (MES), and bathypelagic (BATHY). **(B)** Global geographical distribution of *Nanhaivirus ms29*. The size of the circles represents the relative abundance of *Nanhaivirus ms29*.

The biogeographical distribution of *Nanhaivirus ms29* was examined in 154 viral metagenomes from five VEZs (Vertical Ecological Zones) of the Global Ocean Viromes (GOV2.0) dataset, which included the Arctic (ARC), Antarctic (ANT), temperate and tropical epipelagic (EPI), temperate and tropical mesopelagic (MES), and bathypelagic (BATHY) regions (Gregory et al., 2019). We selected 45 cyanophage sequences from different cyanophage genera under *Kyanoviridae* as references and compared their absolute abundance in GOV2.0 (Figure 5A). In T4-like phages, the abundance of *Nanhaivirus ms29* is relatively low, with the highest abundance observed in the EPI and MES regions. Despite its low abundance, *Nanhaivirus-ms29* was widely distributed across the Indian, Pacific, Atlantic, Arctic, and Antarctic Oceans, with a higher relative abundance in the Indian Ocean than other regions (Figure 5B). Notably, *Nanhaivirus ms29* was highly abundant in the nearshore waters of the Arabian Sea and the Red Sea compared to other sea areas. Furthermore, *Nanhaivirus ms29* was also detected in the arctic pole.

## 4. Conclusion

Isolating and culturing novel cyanophage infecting major bacterial populations, such as *Synechococcus*, is fundamental for understanding cyanophage taxonomy and the relationship between cyanophage and their hosts. In this study, we isolated a novel *Synechococcus* phage *Nanhaivirus ms29* from the oligotrophic ocean basin in the South China Sea. The morphological, growth, genomic, phylogenetic and biogeographical characteristics of *Nanhaivirus ms29* were described. It has a genome of 178,866 bp with G + C content of 42.5%, which contains 217 potential ORFs and 6 tRNAs. We identified as many as 30 auxiliary metabolic genes (AMGs) that could potentially promote important metabolic processes such as photosynthesis, carbon metabolism, nitrogen metabolism, and phosphorus metabolism, which may play an important role in enhancing cyanophage adaptation and promoting host metabolism in the oligotrophic environment. Based on phylogenetic analysis and comparative genomics, we proposed a novel genus, *Nanhaivirus*, in the family of *Kyanoviridae*.

## Data availability statement

The datasets presented in this study can be found in online repositories. The names of the repository/repositories and accession number(s) can be found in the article/supplementary material.

## Author contributions

TW: investigation, methodology, formal analysis, and writing—original draft. LL, YX, CW, and HS: investigation, software, validation, and visualization. MW: conceptualization and supervision. CG: conceptualization, supervision, funding acquisition, and writing—review and editing. All authors contributed to the article and approved the submitted version.

## Funding

Data and samples were collected onboard of R/V “Tan Kah Kee” implementing the open research cruise NORC2021-06 supported by NSFC Shiptime Sharing Project (project number: 42049906). This study was supported by National Natural Science Foundation of China (nos. 42176149, 41906126, 41976117, and 42120104006).

## Conflict of interest

The authors declare that the research was conducted in the absence of any commercial or financial relationships that could be construed as a potential conflict of interest.

## Publisher’s note

All claims expressed in this article are solely those of the authors and do not necessarily represent those of their affiliated organizations, or those of the publisher, the editors and the reviewers. Any product that may be evaluated in this article, or claim that may be made by its manufacturer, is not guaranteed or endorsed by the publisher.

## Footnotes

^1^ http://rast.nmpdr.org/

^2^ http://blast.ncbi.nlm.nih.gov/

^3^ http://pfam.xfam.org/

^4^ https://genome.jp/kegg/

^5^ https://itol.embl.de/

^6^ https://github.com/wwood/CoverM
